# Supplementary material for: Technical report: Targeted proteomic analysis reveals enrichment of atypical ubiquitin chains in contractile murine tissues
Source: J Proteomics. 2020 Oct 30;229:103963. doi: 10.1016/j.jprot.2020.103963 (PMC7567960; doi:10.1016/j.jprot.2020.103963)
Supplement: Supplementary file 1 — Supplementary Table S1. Peptides used for absolute quantification of ubiquitin chain types by Ub-AQUA-PRM analysis. Supplementary Table S2. Multi-step gradient used for separating ubiquitin peptides during Ub-AQUA-PRM analysis. [file mmc8.docx]

**Supplementary Table S1.** Peptides used for absolute quantification of ubiquitin chain types by Ub-AQUA-PRM analysis.

| **Sequence** | **m/z** | **Z** | **RT (min)** | **NCE (%)** | **Transitions** |
| --- | --- | --- | --- | --- | --- |
|  |  |  |  |  |  |
| **GG**M(ox^2^)QIFVK[^13^C_6_^15^N_2_] | 460.2435 | 2+ | 6.7 | 20 | y3^+^, y5^+^, y6^+^, y4^+^, y2^+^ |
| **GG**M(ox^2^)QIFVK | 456.2364 | 2+ | 6.7 | 20 | y3^+^, y5^+^, y6^+^, y4^+^, y2^+^ |
| M(ox^2^)QIFVK(**GG**)TLTGK[^13^C_6_^15^N_2_] | 710.3914 | 2+ | 7.2 | 24 | y8^+^, y6^+^, y9^+^, y7^+^, y5^+^ |
| M(ox^2^)QIFVK(**GG**)TLTGK | 706.3843 | 2+ | 7.2 | 24 | y8^+^, y9^+^, y6^+^, y7^+^, y5^+^ |
| TLTGK(**GG**)TITLEVEPSDTIENVK[^13^C_6_^15^N_2_] | 804.0983 | 3+ | 9.3 | 20 | y9^+^, y9^2+^, y10^+^, y19^2+^, y11^+^ |
| TLTGK(**GG**)TITLEVEPSDTIENVK | 801.4269 | 3+ | 9.3 | 20 | y9^+^, y9^2+^, y10^+^, y19^2+^, y11^+^ |
| TITLEVEPSDTIENVK(**GG**)AK[^13^C_6_^15^N_2_] | 1055.061 | 2+ | 9 | 22 | y11^+^, y12^+^, y13^+^, y3^+^, y14^+^ |
| TITLEVEPSDTIENVK(**GG**)AK | 1051.054 | 2+ | 9 | 22 | y11^+^, y12^+^, y13^+^, y3^+^, y14^+^ |
| AK(**GG**)IQDK[^13^C_6_^15^N_2_] | 412.7394 | 2+ | 5.7 | 26 | y4^+^, y2^+^, y3^+^, y5^+^, y5^2+^ |
| AK(**GG**)IQDK | 408.7323 | 2+ | 5.7 | 26 | y4^+^, y2^+^, y3^+^, y5^+^, y5^2+^ |
| IQDK(**GG**)EGIPP[^13^C_5_^15^N]DQQR[^13^C_6_^15^N_4_] | 551.9536 | 3+ | 6.3 | 22 | y6^2+^, y6^+^, y5^+^, y5^2+^, y3^+^ |
| IQDK(**GG**)EGIPPDQQR | 546.6129 | 3+ | 6.3 | 22 | y6^2+^, y6^+^, y5^+^, y5^2+^, y3^+^ |
| LIFAGK(**GG**)QLEDGR[^13^C_6_^15^N_4_] | 735.9006 | 2+ | 7.3 | 24 | y8^+^, y10^+^, y10^2+^, y9^+^, y2^+^ |
| LIFAGK(**GG**)QLEDGR | 730.8964 | 2+ | 7.3 | 24 | y8^+^, y10^+^, y10^2+^, y9^+^, y2^+^ |
| TLSDYNIQK(**GG**)ESTLHLVLR[^13^C_6_^15^N_4_] | 752.0737 | 3+ | 9.2 | 20 | y16^2+^, y13^2+^, y15^2+^, y14^2+^, y11^2+^ |
| TLSDYNIQK(**GG**)ESTLHLVLR | 748.7376 | 3+ | 9.2 | 20 | y16^2+^, y13^2+^, y15^2+^, y14^2+^, y11^2+^ |
| ESTESTLHLVLR[^13^C_6_^15^N_4_] | 539.3182 | 2+ | 7.2 | 28 | y5^+^, y4^+^, y3^+^, y5^2+^, y6^+^ |
| ESTESTLHLVLR | 534.314 | 2+ | 7.2 | 28 | y5^+^, y4^+^, y3^+^, y5^2+^, y6^+^ |
| TITTITLEVEPSDTIENVK[^13^C_6_^15^N_2_] | 898.4744 | 2+ | 9.5 | 20 | y9^+^, y10^+^, y11^+^, y12^+^, y14^+^ |
| TITTITLEVEPSDTIENVK | 894.4673 | 2+ | 9.5 | 20 | y9^+^, y10^+^, y11^+^, y12^+^, y14^+^ |

*Ox^2^ = sulfone version of methionine

**Supplementary Table S2.** Multi-step gradient used for separating ubiquitin peptides during Ub-AQUA-PRM analysis.

| **Time (min)** | **%B** |
| --- | --- |
| 0 | 0 |
| 4 | 0 |
| 4.25 | 7 |
| 5 | 12 |
| 6 | 15 |
| 7 | 16 |
| 8 | 24 |
| 8.25 | 35 |
| 8.5 | 90 |
| 8.75 | 90 |
| 9 | 0 |
| 10 | 0 |
